# Perinatal Cerebellar Injury in Human and Animal Models

**DOI:** 10.1155/2012/858929

**Published:** 2012-02-23

**Authors:** Valerie Biran, Catherine Verney, Donna M. Ferriero

**Affiliations:** ^1^Departments of Neurology and Pediatrics, Newborn Brain Institute, University of California San Francisco, San Francisco, CA 94143, USA; ^2^U676 Inserm, Paris, France; ^3^Faculté de Médecine Denis Diderot, Université Paris 7, 75010 Paris, France; ^4^Assistance Publique Hôpitaux de Paris, Service de Pédiatrie et Réanimation Néonatales, Hôpital Robert Debré, 48 Baulevard Sérurier, 75019 Paris, France; ^5^PremUP, Paris, France

## Abstract

Cerebellar injury is increasingly recognized through advanced neonatal brain imaging as a complication of premature birth. Survivors of preterm birth demonstrate a constellation of long-term neurodevelopmental deficits, many of which are potentially referable to cerebellar injury, including impaired motor functions such as fine motor incoordination, impaired motor sequencing and also cognitive, behavioral dysfunction among older patients. This paper reviews the morphogenesis and histogenesis of the human and rodent developing cerebellum, and its more frequent injuries in preterm. Most cerebellar lesions are cerebellar hemorrhage and infarction usually leading to cerebellar abnormalities and/or atrophy, but the exact pathogenesis of lesions of the cerebellum is unknown. The different mechanisms involved have been investigated with animal models and are primarily hypoxia, ischemia, infection, and inflammation Exposure to drugs and undernutrition can also induce cerebellar abnormalities. Different models are detailed to analyze these various disturbances of cerebellar development around birth.

## 1. Introduction

Premature birth is a significant risk factor for adverse motor, coordination, cognitive, and behavioral outcomes in survivors [1]. The prevailing brain pathology in very preterm infants is diffuse white matter injury in the cerebral hemispheres [2]. In addition, a consistent pattern of regionally specific long-term volume reduction and abnormalities in cortical and deep grey matter structures in ex-preterm infants is now recognized [3, 4]. Injury and impaired development of the cerebellum, involving both white matter and grey matter components as a complication of premature birth, are also becoming increasingly recognized with advanced neonatal brain imaging [5–11].

Survivors of preterm birth demonstrate a constellation of long-term neurodevelopmental deficits, many of which are potentially related to cerebellar injury, including impaired motor functions such as hypotonia, fine motor incoordination, ataxia, and impaired motor sequencing [12, 13]. Cerebellar injury has also been implicated in cognitive, social, and behavioral dysfunction among older patients [14, 15] and may contribute to the long-term cognitive, language, and behavioral dysfunction seen among 25% to 50% formerly preterm infants [16–19].

The cerebellum is considered particularly vulnerable in the newborn human because of its very rapid growth at that time, a period comparable in the developing animal. The concept of a particular vulnerability of the cerebellum during its phase of rapid growth is documented in experimental models of undernutrition, glucocorticoid exposure, and X-irradiation [20–22].

This article reviews the morphogenesis and histogenesis of the human and rodent developing cerebellum, and its more frequent injuries in preterm. Most cerebellar lesions are cerebellar hemorrhage and infarction usually leading to cerebellar abnormalities and/or atrophy but the exact pathogenesis of lesions of the cerebellum is unknown. The different mechanisms involved, infection, inflammation, hypoxia, ischemia, exposure to drugs, and undernutrition, have been investigated with animal models. These models will be detailed to analyze the disturbance of cerebellar development around birth.

## 2. Review of Cerebellar Histology and Development

### 2.1. Cytological Layering and Specific Cellular Organization of the Cerebellar Cortex

**Figure 1:**
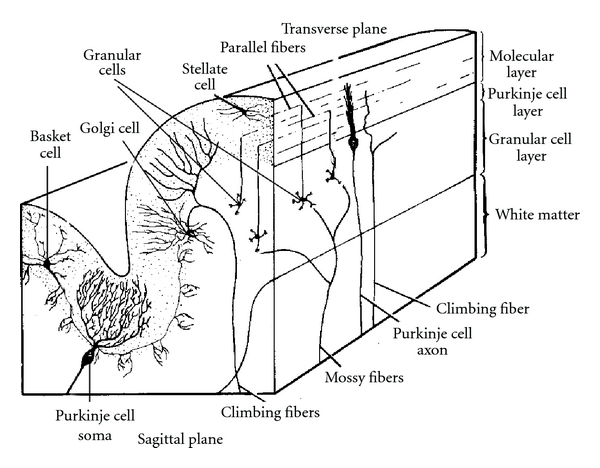
Organization of the mammalian cerebellar cortex in transverse and sagittal planes. Adapted from Brain Res 1981 [79].

The cerebellum is composed of three major histological subdivisions: the cortex, the underlying white matter, and the deep cerebellar nuclei. The basic histological layering of the cerebellar cortex is similar in rodents and primates: the deep granular cell layer, the Purkinje cell layer, and the superficial molecular layer are shown in the simplified schema in coronal and sagittal planes (Figure 1). From the eight classes of cells found in the cerebellar cortex, only the Purkinje cell axons project outside the cortex [23]. The others are local circuit neurons: the granular cells and unipolar brush cells are glutamatergic whereas the others, in particular the stellate, the Golgi, and the basket cells, are GABAergic. The Purkinje cells give rise to the sole output pathway of the cerebellar cortex. The two main afferent pathways conveying information to the cerebellar cortex are the climbing and mossy fibers systems that direct their impulses differently to the Purkinje cells. The climbing fibers originate from the inferior olivary nucleus and established directed synaptic contacts with dendrites of the Purkinje cells. The afferent mossy fibers originate from neuronal populations from various nuclei of the spinal cord, the brain stem, and even the deep cerebellar nuclei. They reach the Purkinje cell indirectly through relay cells, the granular cells via their axonal field, and the parallel fibers [23]. The Purkinje cells are therefore the pivotal elements around which all the cerebellar circuits are organized by receiving information, processing it, and channeling towards efferent pathways.

### 2.2. Connectivity of the Cerebellum

The characteristic neuronal arrangement consists of a strict positioning of neurons and afferent fibers conferring to the cortex a stereotyped three-dimensional geometry [24], which is very helpful to analyze any changes which may occur in the properties of neurons and their connectivity. In addition, the organization of connectivity shows differences in primate versus rodents. The cerebral cortical areas of the forebrain make several axonal connections with the cerebellum via the pallidum, the thalamus, and the pons in mammals. Whereas in humans unilateral and crossed afferents connections running along the superior peduncle in the cerebellum are the most predominant, these corticopontocerebellar projections are bilateral in the rat brain.

### 2.3. Prenatal Development

**Figure 2:**
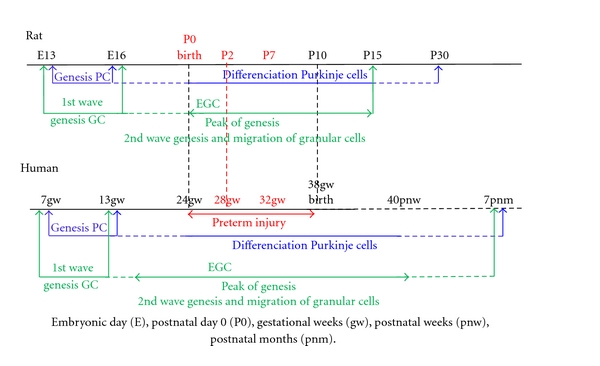
Comparison of timing of development of the Purkinje cells (PC) and granular cells (GC) in the cerebellar cortex in rat and human. EGL: external granular layer. Embryonic day (E), postnatal day 0 (P0), gestational weeks (gw), postnatal weeks (pnw), postnatal months (pnm).

**Figure 3:**
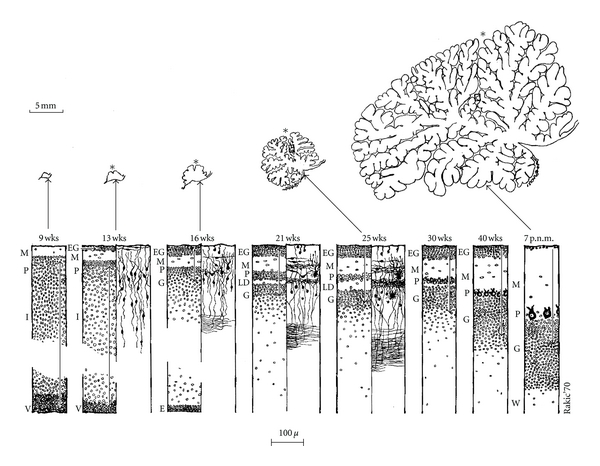
Summary of the main morphogenetic and histogenetic events during development of the human cerebellum from the ninth gestational week (wks) to the seventh postnatal month (p.n.m.) shown in sagittal plane at the level of the primary fissure. E: ependyma, EG: external granular layer, G: Granular layer, I: intermediate layer, L: laminar dissecans, M: molecular layer, P: Purkinje cell layer, V: ventricular zone. W: white matter. The 5 mm scale in the upper corner of the figure show the dramatic increase of the cerebellum primordium especially from the beginning of foliation to 16 wks to 7 pnm. Source: from Brain Res 1973 [28].

Contrary to other regions of the central nervous system (CNS), cerebellar neurons are generated in two germinative neuroepithelia in two waves of proliferation and migration processes. This development occurs in similar order but at different rates in rodents and primates (Figure 2). During the embryonic period in mammals, the cerebellar primordium arises from both mesencephalic and rhombencephalic vesicles in the isthmic area under the control of the isthmic organizer [25]. The first neurons to be generated are the deep nuclear neurons and all the Purkinje cells that migrate immediately after to the cerebellar plate (Figure 3). In parallel, the first granular cell precursors are generated in the rostral rhombic lip (with other neuronal cell populations), and they migrate as precursor granular cells tangentially to cover the superficial zone of the cerebellar plate following a lateromedial and anteroposterior direction (see [23]). They form the extragranular layer (EGL).

### 2.4. Postnatal Development

During postnatal life, the second wave of proliferation occurs in the EGL, the secondary germinal zone giving rise to the granular cells which migrate radially inward to their final destination in the internal granular layer (IGL). The proliferation of granular cells is regulated by Purkinje cells (PC) secreting the Sonic hedgehog signaling factor [23]. In the molecular layer, the onset of synaptic inputs of the axons of the granular cells (parallel fibers) is concomitant with the onset of the final postsynaptic dendritogenesis of the Purkinje cells. The synaptic inputs, essentially from the parallel fibers but also from the climbing fibers, are essential for the achievement of the espalier arrangement of the dendritic trees of the Purkinje cells. In the rat, although the extension of the lateral domain of the dendritic tree of the PC is achieved at postnatal day 15 (P15), its final extension, that is, adult size, is reached at P30. Altman and Bayer [26] described in the rat a caudorostral gradient of development of the cerebellar cortex. In human, the adult number of folia is achieved around two months postnatally [27] and the EGL disappears around the 7th postnatal month [28]. Interestingly, in vivo 3-dimensional volumetric imaging techniques shows, an increase in the cerebellar volume of about 5-fold from 24 to 40 gestational weeks (gw) [29, 30].

## 3. Cerebellar Lesions of the Premature and Term Infants

Lesions such as cerebellar hemorrhage (CBH), infarction, and cerebellar atrophy have been increasingly diagnosed in preterm and term infants using improved neuroimaging techniques [4, 9, 10, 17, 31, 32]. The incidence of these lesions is strikingly dependent on the degree of prematurity. Thus, in the study of Limperopoulos et al. [17], the incidence of lesions in infants <750 g birth weight was 15%, and 2% were seen in those >750 g to 1499 g. The topography of the CBH is primarily focal, unilateral, and within the peripheral parenchyma of the cerebellar hemisphere. Subpial germinal matrix bleeding within the external granular layer may account for some intrahemispheric CBH. The vermis is involved in slightly less than one-third of patients [17]. Cases of vermian hemorrhage represent hemorrhage within the germinal matrix located in the subependymal layer of the roof of the fourth ventricle [33, 34].

CBH may occur concomitantly with cerebral lesions such as hemorrhagic parenchymal infarction, intraventricular hemorrhage with dilatation, and periventricular leukomalacia. In these last cases, premature infants at term-equivalent age have reduced cerebellar volume. This reduction may be due to a primary cerebellar injury that is not detectable by MR imaging at term-equivalent age or due to Wallerian degeneration secondary to cerebral lesions. Cerebellar atrophy is usually focal in the unilateral supratentorial lesions and often generalized in the bilateral cerebral lesions [3]. These data suggest important insights into the highly integrated anatomic and functional integrations between the cerebrum and the cerebellum during development, such as trophic transsynaptic effects along the corticopontocerebellar pathway.

The neuropathological basis of the decreased cerebellar volume remains to be elucidated. In preterm of 32 gestational week (gw), neuronal loss and gliosis were detected in dentate nucleus, cerebellar cortex, or the brain stem cerebellar relay nuclei, basis pontis, and inferior olive, in only 5% to 15% of infants, in particularly in presence of leukomalacia [6].

The possibility that cerebellum atrophy in premature infants may be related to adverse blood product as hemosiderin deposit following hemorrhage has been suggested by Messerschmidt and colleagues [5, 19, 33]. Tam et al. [4] found that more severe supratentorial intraventricular hemorrhage (IVH) was associated with slower growth of cerebellar volumes. No changes in volumes were found with IVH at 30 weeks postmenstrual age (95% CI 26–33 weeks), but volumes by 40 weeks were 1.4 cm^3^ lower in premature infants with grade 1-2 IVH and 5.4 cm^3^ lower with grade 3-4 IVH. The same magnitude of decreased volume was found whether the IVH was ipsilateral or contralateral. No association was found with severity of white matter injury (*P* = 0.3).

Whether these blood products are crucial or not in the onset of the cerebellar lesion remain unclear (see [33]). Early effects of decreased cerebellar volume associated with supratentorial IVH in either hemisphere may be a result of concurrent cerebellar injury or direct effects of subarachnoid blood on cerebellar development.

 Preterm delivery associated to other adverse insults could disrupt the developmental program of the cerebellum. A recent postmortem study on premature infants who had survived in an exutero environment reports cerebellar abnormalities in the development of granular cells which parallel a decrease of Sonic hedgehog in the Purkinje cell layer [35].

In fact the pathogenesis of lesions of the cerebellum is multifactorial. Univariate analyses identified maternal, intrapartum, and early postnatal hemodynamic risk factors; multivariate regressions indicate that emergent caesarian section, patent ductus arteriosus, and lower 5-day minimum pH independently increased the odds of cerebellar hemorrhage [17]. Different mechanisms appear plausible to explain the disturbance of cerebellar development after premature birth. The correlation of lesions of the cerebellum in preterm with animal models can highlight the precise pathophysiology of these lesions. 

## 4. Mechanisms of Cerebellar Lesions in Preterm: Correlations with Animal Models

### 4.1. Hypoxia-Ischemia

#### 4.1.1. Magnetic Resonance Imaging Studies

The damaging influence of hypoxia or hypoxia-ischemia to the cerebellar underdevelopment is suggested by the strong correlation of the cerebellar abnormality with MRI-demonstrated supratentorial injury [3, 4, 9, 11, 32]. In the largest reported MRI series of very preterm and preterm, a decrease in cerebellar volume at term equivalent age correlated with decreasing gestational age [30]. In the pathology of preterm infants, neuronal loss detected in the cerebellum and related brain stem nuclei was essentially confined to the infants with periventricular leukomalacia (25% to 30% of infants) [6]. Primary impaired cerebellar development of different origins, such as hypoxia-ischemia, has most often consisted of bilateral, generally symmetric deficits in the cerebellar hemispheric volumes [33]. On the other hand, a recent MRI study suggested that unilateral injury confined to the preterm cerebral hemisphere was associated with a significantly decreased volume of the contralateral cerebellar hemisphere [3]. These data suggest that two main mechanisms might induce the impaired cerebellar development of the premature brain: either a direct effect on the development of the cerebellar cortex or remote effects operating via trophic transsynaptic interaction between the telencephalic leukomalacia and the developing cerebellum via the corticopontocerebellar pathway. On the other hand a recent study by Tam et al. showed that cerebral white matter injury did not correlate with reductions in cerebellar volume [4].

### 4.2. Rodent Models at Postnatal Day 2

To address this question a model of the preterm human in neonatal rat pups was developed on postnatal day 2 (P2) which is comparable to 28 weeks of gestation in the human (Figure 2), when the cerebellar cortex is the most vulnerable to insult (see Section 2.4). As mentioned previously, the second wave of neuronal cerebellar proliferation plays a key role in the organization of the cerebellar cortex. In a previous study we demonstrated that global hypoxic injury or forebrain hypoxia-ischemia at P2 in rat pups produce dramatic cellular damages in the cerebellar cortex [36]. Interestingly, the addition of forebrain ischemia does not increase the huge cellular damage obtained following hypoxia which contradict the afore mentioned hypothesis about a possible correlation between cerebral and cerebellum [3]. Our results showing neuronal and white matter damage in both cerebellar hemispheres following hypoxia alone suggest that systemic hypoxia could adversely affect the developing cerebellum independent of its connections at this developmental stage. The defect in myelination detected following hypoxia alone is even more severe than that following hypoxia-ischemia. The lack of volume loss detected at P21 indicates that there can be significant cellular injury followed by gliosis and postlesional plasticity with axonal and dendritic growth. The presence of increased density of GFAP-positive cells and microglial activation in the white matter and cerebellar cortex of both hypoxic and hypoxic-ischemic injured rats supports a pathological event directly affecting the survival and/or maturation of neurons and preoligodendrocytes. These findings may explain some neurodevelopmental abnormalities seen in preterm babies even in the absence of gross cerebellar volume reduction.

### 4.3. Rodent Models at Postnatal Day 7

Following hypoxia-ischemia, selective vulnerability of different regions of the brain depends on its maturity and on the severity of the insult [37]. In the P7 hypoxic-ischemic model (Vannucci model) equivalent of human injury at 32–36 weeks of gestation (Figure 2) the areas with higher metabolism such as the cerebral cortex, hippocampi, and deep gray nuclei suffer the most after initial ischemic injury. Histological brain damage is generally confined to the cerebral hemisphere ipsilateral to the arterial occlusion, and consists of selective cell death or infarction and delayed neurodegeneration depending on the duration of the systemic hypoxia [38–40].

Other studies using perinatal hypoxia-ischemia have shown that cell death occurs in brain regions that are not directly affected by the ischemia, such as cerebellum [39, 41, 42] suggesting that neuronal connectivity may play a role in neurodegeneration following hypoxia-ischemia to the immature brain (P7 age). Taken together, these findings may reveal the connection networks which could exist between the damaged forebrain and cerebellum in the developing mammal brain. In rodent models, forebrain hypoxia-ischemia may affect differently the corticopontocerebellar connections according to the age of the insult. As aforementioned, these lesions may not occur at P2 but could be present at P7. In human, the activity in the ipsilateral pons, and also the contralateral cerebellar cortex, is a phenomenon known as crossed cerebellar diaschisis [43]. Limperopoulos et al. [3] showed that unilateral injury confined to the preterm cerebral hemisphere was associated with a significantly decreased volume of the contralateral cerebellar hemisphere, and that these effects were evident as early as term gestational age equivalent. Limperopoulos et al. [3] hypothesized that the corticopontocerebellar connections are involved in cerebellar damage. More studies are necessary to confirm this hypothesis.

### 4.4. Infection and Inflammation

A strong relation of maternal intrauterine infection with systemic fetal inflammation or of postnatal neonatal infection with systemic inflammation and the occurrence of periventricular leukomalacia is well documented [44, 45]. White matter damage, astrocytosis, and cytokine activation have been demonstrated in experimental model of intrauterine infection, all of which are capable of leading to delays in brain development [46, 47]. The cerebellum is particularly vulnerable to infections insults since it is not fully developed until after birth in both humans and rodents [22, 33]. Due to the nearly 5-fold increase in growth in the cerebellum in the last trimester of pregnancy, intrauterine infection, or activation of the fetal immune system could cause irreparable damage to this structure [29]. Experimental studies of E. *coli* injection administered at gestational day 17 in rats decreased Purkinje cell density and volume [48]. The decrease in calbindin in Purkinje cells is also accompanied by impairment in motor coordination and balance in rats from the early postnatal period through adulthood [49]. In fetal preterm sheep, exposure to bacterial endotoxin (lipopolysaccharide; LPS) cause a diffuse pattern of cerebellar white matter damage [50, 51]. Injury to the cerebellar white matter involves diffuse loss of oligodendrocytes, associated with apoptotic and/or inflammatory processes, which is similar to the white matter injury observed in the forebrain of preterm infants [2] and in experimental immature animal models [52].

Human cytomegalovirus infection of the developing central nervous system (CNS) is also a major cause of neurological damage in newborn. To investigate the pathogenesis of this human infection, animal models of virus infection of the CNS are associated with a delay of the morphogenesis of the cerebellum [53, 54]. The defects in cerebellar development in infected animals located in the cerebellar cortex are correlated temporally with virus replication and CNS inflammation, spatially unrelated to foci of virus-infected cells. CMV-infected cells are more prevalent in the Purkinje cell layer than in the mitotic granule cell layer [55]. In an animal model of lymphocytic choriomeningitis virus [56], there is selective infection of several neuronal populations and in focal pathological changes. Astrocytes and Bergmann glia cells are the first cells of the brain parenchyma infected with LCMV and the virus spreads across the brain principally via contiguous glial cells. The virus then spreads from glial cells into neurons. LCMV infects neurons in only four specific brain regions: the cerebellum, olfactory bulb, dentate gyrus, and periventricular region. The cerebellum undergoes an acute and permanent destruction while the olfactory bulb is acutely hypoplastic but recovers fully with age. Neurons of the dentate gyrus are unaffected in the acute phase but undergo a delayed-onset mortality. In contrast, the periventricular region has neither acute nor late-onset cell loss.

Currently, there are no direct data on the role of infection/inflammation in the genesis of cerebellar abnormality of the human premature infant.

### 4.5. Drug Exposure

Maternal exposure to nicotine, cocaine, and ethanol during pregnancy is known to be a significant contributor to neurobehavioral deficits in the offspring [57], and specific studies of the cerebellum in this context are of particular interest.

In animal studies, nicotine treatment via injection during gestation has been shown to produce episodic hypoxia in the developing fetus. Abdel-Rahman et al. [58] evaluated the neurotoxicity in the offspring at pubertal stage of the development following continuous maternal exposure to nicotine via infusion during the gestation period. Histopathological findings showed a significant decrease in the surviving Purkinje neuronal cells in the cerebellum and CA1 subfield of hippocampus in the offspring on postnatal day 30 and 60. These pathological findings suggest that the deficits in the cerebellum could persist longer than other brain regions [59]. Furthermore, there was a significant increase in GFAP immunostaining in both cerebellar white matter and granular cell layer as well as the CA1 subfield of the hippocampus.

The mechanisms which alcohol induces their effects on development are unknown. A study by Bonthius et al. showed that gestational exposure to ethanol in a nonhuman primate species induced permanent doserelated deficits in the number of cerebellar Purkinje cells. The number of Purkinje cells and their linear frequencies were significantly reduced in the alcohol-treated subjects, and the deficits were dose-dependent. The findings suggest that alcohol-induced reduction in neuronal number may be an important factor underlying the CNS dysfunction in fetal alcohol syndrome [60].

### 4.6. Glucocorticoid Exposure

The developmental effects of glucocorticoids on the cerebellum are an important area of study as the cerebellum has the highest levels of glucocorticoid receptors in the brain, localized in the external granular layer [61, 62]. Studies in human preterm newborns reveal adverse effects of postnatal dexamethasone therapy on brain development, including decreased cerebral and cerebellar tissue volumes [63]. In a recent study by Tam et al. [11], preterm newborns were prospectively studied with serial MRI examinations near birth and again near term-equivalent age. Adjusting for relevant clinical factors, antenatal betamethasone was not associated with changes in cerebellar volume but postnatal exposure to clinically routine doses of hydrocortisone or dexamethasone was associated with impaired cerebellar growth. Cerebral growth was not affected [11, 64].

Animal models demonstrate reduced preterm cerebellar growth after exposure to all glucocorticoids including hydrocortisone, dexamethasone, and corticosterone [62, 65, 66]. In the developing cerebellum, glucocorticoids cause neuronal apoptosis and inhibit proliferation of immature granule neuron precursors. However, although 11-*β*-hydroxysteroid dehydrogenase type 2 is capable of degrading hydrocortisone but not dexamethasone, both glucocorticoids result in injury of the external granular layer in wild-type animals. This is suggested by rodent models showing similar effects of corticosterone (a known substrate of 11-*β*-hydroxysteroid dehydrogenase type 2) and dexamethasone on granule cell apoptosis with acute glucocorticoid exposure and inhibition of cell proliferation with chronic exposure [67]. Heine et al. [68] recently showed that systemic administration of a small-molecule agonist of the Sonic hedgehog-Smoothened pathway (SAG) prevents neurotoxic effects of GCs susceptible to metabolism by the enzyme 11*β*-hydroxysteroid dehydrogenase type 2, but that it does not interfere with beneficial effects of glucocorticoids on lung maturation. These findings suggest the potential of a small molecule agonist of Smoothened as a neuroprotective agent in the setting of glucocorticoid-induced neonatal cerebellar injury.

### 4.7. Undernutrition

In the study of Limperopoulos et al. [30], cerebellar volumes were significantly associated with head circumference and weight at term-equivalent age MRI. Insufficient postnatal catch-up growth in preterm infants has been significantly associated with adverse neurodevelopmental outcome [69, 70]. These data suggest that impaired postnatal growth may be an important marker of impaired central nervous system integrity and, in particular, deficient cerebellar growth at term. However, prospective studies in preterm (less than 30 weeks' gestation age) suggest that suboptimal early nutrition in preterm infants can have a permanent effect on their cognitive function, emphasising the potential importance of dietary management decisions in this population [71, 72].

Many experimental data show that during its phase of rapid growth, the cerebellum is especially vulnerable to undernutrition [21, 22, 73]. Rees et al. [74] showed no overt signs of damage in sheep brains and cerebellum from intrauterine growth restricted (IUGR) fetuses; however, morphological analysis demonstrated subtle but important alterations in connectivity and function. In the cerebellum, the most important finding was a 20% reduction in the area of arborization of Purkinje cell dendrites and a 26% decrease in the total number of dendritic spines. As spines are the sites of synaptic apposition, synaptic input to Purkinje cells are reduced with a possible alteration in cerebellar function [74–76]. Restricted cerebellar growth and differentiation is also shown in studies of placental insufficiency in fetal sheep and guinea pigs [77, 78].

## 5. Conclusion

Cerebellar injury and disorders of development are now a recognized problem in preterm infants; these data suggest a potential pathological role in the long-term cognitive, behavioral and motor deficits associated or not with brain injury. The precise pathophysiology of cerebellar injury remains unknown in preterm infants, and it is necessary to interrogate animal models to unravel the main mechanisms. In parallel, sophisticated pathological data on premature cerebellum are necessary to analyze specific human features. In addition, pathological investigations associated with MRI studies on the same cerebellum are an essential step to define biomarkers necessary to improve the prognosis of cerebellar damage in preterm infants.
